# Anatomical Abnormalities in Gray and White Matter of the Cortical Surface in Persons with Schizophrenia

**DOI:** 10.1371/journal.pone.0055783

**Published:** 2013-02-13

**Authors:** Tiziano Colibazzi, Bruce E. Wexler, Ravi Bansal, Xuejun Hao, Jun Liu, Juan Sanchez-Peña, Cheryl Corcoran, Jeffrey A. Lieberman, Bradley S. Peterson

**Affiliations:** 1 Division of Child and Adolescent Psychiatry, The New York State Psychiatric Institute, Columbia College of Physicians and Surgeons, New York, New York, United States of America; 2 Yale University School of Medicine, New Haven, Connecticut, United States of America; 3 Department of Psychiatry, The New York State Psychiatric Institute, Columbia College of Physicians and Surgeons, New York, New York, United States of America; Catholic University of Sacred Heart of Rome, Italy

## Abstract

**Background:**

Although schizophrenia has been associated with abnormalities in brain anatomy, imaging studies have not fully determined the nature and relative contributions of gray matter (GM) and white matter (WM) disturbances underlying these findings. We sought to determine the pattern and distribution of these GM and WM abnormalities. Furthermore, we aimed to clarify the contribution of abnormalities in cortical thickness and cortical surface area to the reduced GM volumes reported in schizophrenia.

**Methods:**

We recruited 76 persons with schizophrenia and 57 healthy controls from the community and obtained measures of cortical and WM surface areas, of local volumes along the brain and WM surfaces, and of cortical thickness.

**Results:**

We detected reduced local volumes in patients along corresponding locations of the brain and WM surfaces in addition to bilateral greater thickness of perisylvian cortices and thinner cortex in the superior frontal and cingulate gyri. Total cortical and WM surface areas were reduced. Patients with worse performance on the serial-position task, a measure of working memory, had a higher burden of WM abnormalities.

**Conclusions:**

Reduced local volumes along the surface of the brain mirrored the locations of abnormalities along the surface of the underlying WM, rather than of abnormalities of cortical thickness. Moreover, anatomical features of white matter, but not cortical thickness, correlated with measures of working memory. We propose that reductions in WM and smaller total cortical surface area could be central anatomical abnormalities in schizophrenia, driving, at least partially, the reduced regional GM volumes often observed in this illness.

## Introduction

Although reduced whole brain volume, reduced gray matter (GM) volumes and white matter (WM) abnormalities have been described in schizophrenia, the exact nature of these anatomical abnormalities and the relationship of disturbances in one tissue type to those in another remain elusive. Findings in GM and WM have been inconsistent across studies, in part because both tissue types have seldom been assessed simultaneously, and when they have, measures have been of relatively large regions-of-interest (ROIs), the use of which risks averaging out volumetric abnormalities if the region contains a combination of anatomically normal and pathological tissues. Nevertheless, ROI studies - either those examining larger regions or those using more parcellated subdivisions - have revealed discrete volumetric abnormalities, especially in frontal, cingulate and temporal areas [1]. Additionally, whole-brain approaches such as voxel based morphometry, have identified volumetric deficits in frontal WM [2] and within the left temporal regions [3]. Finer-grained approaches to spatial localization of anatomical abnormalities, such as tensor-based deformation measures of cortical thickness and morphological assessment of the brain surface [4] have further expanded our understanding of anatomical abnormalities in schizophrenia, revealing cortical thinning in heteromodal association cortices [5] as well as brain surface contraction [6] and altered frontal gyrification patterns [7]. Notwithstanding the increasing complexity of the anatomical measures used in these imaging studies, few have assessed the differing aspects of GM and WM anatomy simultaneously. A holistic understanding of how these differing components of brain volumes contribute to the volumetric abnormalities observed in schizophrenia is critically important. Cortical surface area and cortical thickness represent different features of brain anatomy and are most likely the products of distinct genetic influences [8], [9]. According to the radial unit hypothesis [10], cortical surface area is driven by the number of columnar units, whereas cortical thickness is determined by the number of cells within a column. Some have suggested that postnatal developmental events affecting GM are preferentially reflected in cortical thickness, although this remains largely speculative [11]–[16].

Building on the extensive anatomical literature in schizophrenia, our study aims to provide a more refined characterization of GM and WM volume abnormalities in schizophrenia. We sought to clarify whether local volumetric reductions of brain tissue along the surface of the brain are associated with reduced local cortical thickness or instead with local volume reductions of the underlying white matter. We also wished to clarify whether reductions of the total surface area of the cortical mantle and of the underlying WM could be driving the regional GM volume reductions frequently observed in schizophrenia. Finally, we aimed to correlate these morphological variables with clinical symptoms and working memory measures, in order to determine whether distinct aspects of the illness were associated with distinct features of brain anatomy.

We studied the fine-grained morphological features of the brain surface, underlying WM surface, and cortical thickness in 76 persons with schizophrenia and 57 age-matched healthy controls using the techniques of tensor-based deformation [4], [17]. Surface morphology permits spatially precise, fine-grained assessments of the shape of the brain surface in which increases or decreases in local volumes of the brain tissue along the brain surface can derive, respectively, from either increases or decreases in local thickness of the underlying cortex or from greater or smaller local volumes along the underlying white matter. These techniques thereby help to obviate the problems posed by use of large ROIs in conventional volumetry, in which localized volume increases and decreases within the same ROI could tend to cancel each other and go undetected.

We hypothesized that abnormalities of brain tissue along the brain surface would correspond to underlying reductions of WM. We based this hypothesis on our prior findings of reductions in WM regional volumes in this sample [18], as well as on emerging evidence suggesting WM reductions in schizophrenia [19]. Informed by a large number of prior imaging, neuropathological, and clinico-pathological observations, we predicted that abnormalities in cortical thickness would be located primarily within limbic and perisylvian cortices, or in the WM underlying those regions.

We also hypothesized that the generalized regional GM volume reductions we previously observed could be explained by a combination of reduced thickness and a reduction in the area of the cortical surface, as previously reported by others [20]. Furthermore, we expected that the contraction of cortical surface area would be accompanied by reduction in the area of the WM underlying the cortical mantle. Second, because of our prior observations [18] as well as of evidence that WM disruptions correlate with performance on verbal working memory [21], we hypothesized that working memory, as specifically measured by the Serial Position Task (SPT) would be correlated with anatomical features of WM rather than GM.

Although associations with symptoms have been reported more frequently with GM [22] rather than with WM abnormalities [23], we expected that symptoms would correlate with anatomical features of the WM. Disruptions in WM connections among brain areas have been posited as the neuroanatomical basis for symptoms and cognitive dysfunction in schizophrenia [19] and studies in functional and structural connectivity [24]–[26] seem to support the idea that schizophrenia could be an illness of disordered connectivity [27], [28]. Given that connections among different brain areas are provided by WM tracts, we hypothesized that the severity of both positive and negative symptoms would correlate with features of WM anatomy.

## Methods

### Sample

Seventy-six symptomatic but stable outpatients who met DSM-IV criteria for schizophrenia, and 57 healthy controls, participated after providing written informed consent. A more detailed description of this sample (Table 1), including neuropsychological and clinical characterization, is provided elsewhere [18]. All but three patients had been in treatment more than five years, most had been hospitalized more than three times (none within three months of the study), none had abused substances within the last 60 days, and all had been on their current medications for at least 30 days. Healthy controls were without history of Axis I disorders or neurologic illness. Length of illness was a best-estimate judgment based on the patient’s report of the first appearance of symptoms or functional compromise related to aspects of schizophrenia (e.g., delusional thinking, paranoid fears, hallucinations, social withdrawal, decline in school performance), first hospitalization, and history of illness as documented in reports related to initial hospitalizations. When insufficient information was available, no duration was assigned.

**Table 1:** Demographic and Clinical Characteristics of Study Participants.

Characteristics	Patients (n = 76)	Healthy Controls (n = 57)	df	F/T or χ^2^	p-value
Sex M/F (N)	51/25 (n = 76)	28/29 (n = 57)	1	4.37	0.04*
Age at MRI, y	41.57 (SD 9.03) (n = 76)	38.36 (SD 11.85) (n = 57)	131	5.6/1.7	0.08
Height, inches	68.19 (SD 4.07) (n = 75)	67.28 (SD 4.49) (n = 54)	127	1.3/1.14	0.25
Handedness, R/L/A (N)	69/7/0 (n = 76)	56/1/0 (n = 57)	1	3.2	0.07
Education, y	12.97 (SD 2.3) (n = 71)	15.9 (SD = 4.6) (n = 44)	113	9.10/−4.50	<0.001*
Duration of Illness, y	17.64 (SD 8.47) (n = 70)	N/A			
Clorphromazine Equivalents (mg/day)	682.22 (SD 613.35) (n = 55)	N/A			
*PANSS Scores*					
Positive	15.25 (SD 6.20) (n = 52)				
Negative	15.77 (SD 5.45) (n = 52)				
General	32.37 (SD 10.68) (n = 52)				
Total	63.38 (SD 18.81) (n = 52)				
*Ethnicity*					
Minority/Caucasian (N)	32/44	8/49	1	12.2	0.001*
*Type of Antipsychotic Medication (N)*					
Atypical	35				
Typical	18				
Atypical +Typical	11				
Clozapine	3				
Atypical+Clozapine	4				
Typical+Clozapine	2				
*Total on Antipsychotic Medications*	73				
Data Unavailable	1				
No Antipsychotic	2				
*Total on Mood Stabilizers*	28				
Valproate	16				
Lithium	7				
Carbamazepine	4				
Lamotrigine	1				
*Total on Antidepressants*	19				

Data are reported as mean (SD). F and T values are reported for independent T tests for means and chi-square values (χ^2^) for nominal data. An asterisk denotes significant p values. N = number.

### Neuropsychological and Clinical Measures

As in our previous study, the patient group was divided into two subgroups based on neuropsychological performance using four Serial Position Tasks (SPT) that assessed verbal and nonverbal working memory using four types of stimuli: words; easily named sounds, such as a ringing telephone; birdsongs; and snowflake designs [18]. One group was considered neuropsychologically impaired on the basis of overall scores on all four tests that were more than 1.0 SD below the mean of healthy controls (NPI, n = 50). The second group was considered neuropsychologically near-normal (NPNN, n = 21) on the basis of overall working memory scores within 0.5 SD of the healthy control mean. These tests were selected for subgroup definition because patients with schizophrenia have been shown in previous studies to perform particularly poorly on these tasks, and because deficits in working memory in general, and verbal memory in particular, are among the most consistent and robust cognitive deficits in schizophrenia [18], [29]. The resulting subgroups were validated by comparing the groups to each other and to healthy subjects on the California Verbal Learning Test and a degraded stimulus CPT, two tests widely used to demonstrate cognitive deficits in people with schizophrenia [30]. In our sample of 76 patients, 5 patients were not classified as either NPNN or NPI because neuropsychological data were not available. Severity of illness was measured using the Positive and Negative Syndrome Scale (PANSS) [31]. PANSS scores were available for 52 of our 76-person patient sample. As reported previously, the two patient subgroups did not differ substantially in medication status or clinical symptoms [18].

### Image Acquisition

Magnetic Resonance Images (MRIs) were acquired using a single 1.5 Tesla GE Signa LS MRI scanner and a 3D spoiled gradient recall pulse sequence (TR = 24 msec, TE = 5 msec, 45° flip, frequency encoding superior to inferior, 256×192 matrix, field of view = 30 cm, 2 excitations, slice thickness = 1.2 mm, no skip, 124 sagittal slices).

### Image Processing

Analyses were performed on Sun Ultra 10 workstations using ANALYZE 8.0 (Rochester, MN), while blind to participants’ characteristics and hemisphere (images were randomly flipped in the transverse plane before preprocessing). Prior to region definitions, large-scale variations in image intensity caused by RF coil and other inhomogeneities were removed [32]. Extracerebral tissues were removed using an isointensity contour function that thresholds cortical GM from overlying cerebrospinal fluid. Connecting dura and fat were removed manually. The dataset was resliced to Talairach standard orientation to correct for residual head rotation, tilt, or flexion/extension. Gray scale values of “pure” representations of cortical GM (the cortical ribbon) and WM were sampled bilaterally in frontal, temporal, occipital, and parietal regions using an 8×8 = 64 pixel array that was sufficiently large to provide statistical stability but small enough to avoid contamination by partial volume effects from other tissue types. These 4 values were averaged for each tissue type. A global threshold, calculated as the average of mean GM and WM values, was invoked to provide an initial rough classification of gray and white matter. This classification was then hand-edited in all 3 views, primarily to eliminate subcortical GM and rims of ventricles (partial volumed WM and ventricular CSF that is labeled as GM in most segmentation algorithms) from the tissue assigned to cortical gray matter. The intraclass correlation coefficient, calculated as a measure of reliability of our segmentation procedures using a 2-way random effects model [33], was 0.98.

### Coregistration

A detailed description of the methods used to analyze surface morphology and their validation is provided elsewhere [17]. Briefly, the random flips of the images (above) were reversed to provide their original correct orientation. Brains were brought into coarse alignment using a similarity registration (translation, rotation, and scaling) that maximized the mutual information of gray scale intensity values of the pixels in each brain with gray scale values in the template brain [4]. Each brain was then warped to the template brain using a high-dimensional, non-rigid warping algorithm based on fluid dynamics, producing a brain that was exactly the same size and shape as that of the template brain, thereby permitting identification of precisely corresponding points on the surfaces of each brain and of the template brain. The warped brains were then unwarped by reversing this high-dimensional, nonlinear warping, bringing along with each surface the labels that identified corresponding points on the template brain. We applied a rigorous two-step procedure to select a brain template that was the closest possible (in terms of least squares error) to the average brain shape in all the healthy controls. First, a preliminary reference was selected as the brain of the healthy control who was as demographically representative as possible of all healthy controls. The brains of all the other controls were then normalized to this preliminary reference. The point correspondences on the brain surfaces were determined and we computed the distance from the template surface for each of the corresponding points on the surfaces of the brains of all other healthy controls. Subsequently, the brain for which all points across its surface were closest, in terms of least square means, to the average of the computed distances was selected as the final template. We used a single representative brain as template rather than an average brain because the use of a single brain, which has sharp borders at the CSF-GM or GM-WM interface, improves the accuracy of registration. Averaging images to generate a template blurs these boundaries, increasing registration errors that make detection of subtle effects across populations more difficult. Furthermore, precise surface morphometry requires a brain with smooth GM and WM surfaces that are devoid of topological defects, which cannot be adequately reconstructed by averaging brains across multiple healthy controls. After unwarping, we calculated the distance of each point on the brain surface of each brain to the corresponding point on the brain surface of the template brain. At a group level this procedure generated, at each point on the brain surface, a set of signed Euclidean distances, which we subjected to statistical modeling. We conducted similar analyses for the WM surface. Indentations or protrusions along the surface of the brain or along the surface of the WM were interpreted as representing greater or smaller local volumes, respectively, of the brain tissue along those surfaces. We displayed statistical results for the brain surface and for cortical thickness on the ICBM (International Consortium for Brain Mapping) high-resolution, single-participant template [34]. The delineation of cortical gyri was performed manually by an expert neuroanatomist, and this 3-D set of labels was mapped onto the brain surface of our template brain to delineate regional boundaries.

### Cortical Thickness

From the coregistered brain of each participant we subtracted its cortical mantle. We then used a three-dimensional morphological operator to distance-transform this brain without the cortex from the coregistered brain of the same participant that contained the cortex [35]. This operation calculated cortical thickness as the smallest distance of each point on the external cortical surface from the outermost surface of the WM in the coregistered brain.

Due to the fact that these thicknesses were measured in template space, their values inherently accounted for generalized scaling effects within the cerebrum, because the brain and its local features, such as cortical thickness, were already scaled during the similarity transformation of that brain to the template. Because cortical thickness was poorly defined for one of the 76 patients, we did not include this subject in our analyses of the cortical thickness and of the WM surface. There was no difference in results for our analyses whether we included this subject or not.

### Surface Area

We computed the total surface area of the cortex for each individual brain coregistered to the template brain, which ensured that the computed measure was appropriately scaled by the ratio of the whole brain volume (WBV) for each individual to that of the template brain. We obtained the area of the surface generated by removing two layers of voxels from the brain to better delineate the sulci, allowing us to include the surface area for the sulci of the tightly packed gyri [36]. To compute the surface area: (1) we multiplied the number of voxels on this eroded surface by the average area of the three faces of each voxel (voxel method), and (2) we applied the method of marching cubes (refer to the Methods S1) to first extract a triangulated surface of the eroded cortical area, and subsequently summed up the area of each triangle to compute the total surface area (triangulation method). In addition, we applied these two methods to compute the total surface area of the subcortical WM for each individual brain coregistered to the template brain.

### Statistical Analysis

Either cortical thickness measures or signed Euclidean distances describing the brain and the WM surfaces for each participant were entered as dependent variables in a multivariable linear regression conducted at each voxel. We covaried for age, age^2^, and gender of the participant, having first ensured the absence of significant interactions among these covariates. We used both age and its quadratic term to control for the linear and quadratic effects of age on volumetric variables. The regression model had the form
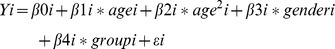
where Y_i_ was either the *signed Euclidean distance* or the *value of cortical thickness* at each point on the cerebral (or white matter) surface of the i^th^ brain, and β_0i_, β_1i,_ β_2i,_ β_3i,_ and β_4i,_ were the intercept and regression coefficients for the linear effect of age, the quadratic effect of age, the effect of gender, and group membership (control or patient), respectively, for participant i. Each regression coefficient was estimated and the p-value for each term was obtained using the T-statistic. In order to determine the relationship between abnormalities along the brain surface and abnormalities of the cortical thickness across the brain, we controlled, at each point of the brain surface, for the underlying cortical thickness by adding cortical thickness as covariate to the above model and using signed Euclidean distances as a dependent variable. Correlation of morphological measures with symptom severity was performed using the following general linear model in the patient group only, calculating the regression term β_5i_ for the term representing the PANSS scores, with scores for negative and positive symptoms entered separately:



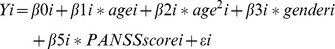



A similar regression model was used to model the effects of overall SPT memory score on anatomical measures (Y_i_). In addition to these main effects, we considered for inclusion in the model all 2-way interactions of diagnostic group, gender, age and minority status. Non-significant terms were dropped from the final model. We did not include in the model the interaction of age and diagnostic group because, though the interaction was significant at a small number of voxels in the WM surfaces of both hemispheres, it was at locations that differed from the locations of voxels where the significant main effects of diagnosis were detected (Fig. S4). Although the main effect of minority status was significant for cortical thickness in the patient group (Fig. S5), we decided not to include minority status in our final model because our results did not differ whether or not we modeled this term. We used a similar regression model to regress cortical surface area or WM surface area onto age, age^2^, gender and diagnostic group. Chlorpromazine equivalents were available for close to three fourths of the sample (n = 55). As a measure of lifetime exposure to antipsychotic medications, we used the scalar product of current chlorpromazine equivalents and length of illness, in a manner similar to previous studies [37]. This regressor was entered as a confounder in the group comparisons, with no change in results for all anatomical measures. Therefore, the variable was not retained in the final model

### Correction for Multiple Comparisons

We corrected for multiple comparisons using the theory of Gaussian Random Fields (GRF) [17]. We color-coded the p-values of the appropriate regression coefficient at each voxel and displayed them across the surface of the template brain. We also conducted exploratory analyses without applying GRF correction. We confirmed our results by applying Bonferroni correction, using a corrected p-value of p<0.05/12 because we performed a total of 12 comparisons when we correlated one neurocognitive measure (overall SPT memory score) and three clinical measures with the 3 morphometric variables (cortical thickness and local volumes along the surfaces of the cortical mantle and of the underlying WM).

## Results

### Diagnosis-specific Effects

**Figure 1:**
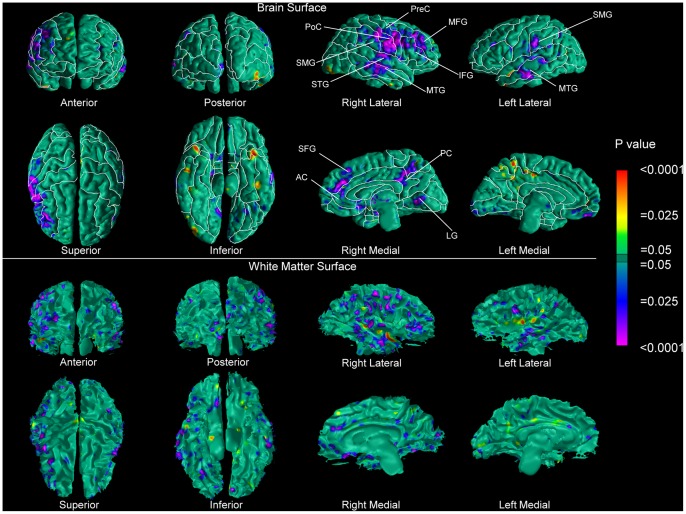
Maps of Group Differences in Local Volumes along the Surface of the Brain and of the Underlying WM in Persons with Schizophrenia Compared with Healthy Controls. At each point on the brain or on the WM surface, the statistical significance of differences in local volumes between patients (n = 76) and healthy controls (n = 57) is color-coded. Warm colors (yellow, orange and red) represent significantly greater local volumes of brain tissue along the surface of the brain or of WM tissue along the underlying WM surface in the schizophrenia group. Cooler colors (blue and purple) represent reduced local volumes of brain tissue along the surface of the brain or of WM tissue along the underlying WM surface in that group. The color bar indicates the color-coding of p values for testing of statistical significance at each point. Brain and WM surfaces were rescaled for whole brain size and the statistical models accounted for age, age^2^ and the gender of all participants. P values are thresholded at P<0.05 with corrections for multiple statistical comparisons using the theory of Gaussian Random Fields (GRF) on a 2D manifold. P values are displayed onto the ICBM brain surface of the template brain (in green) and onto the WM surface of the reference subject. More specifically, abnormalities in WM surface, brain surface, and cortical thickness are overlaid on the same reference brain. However, while brain surface and cortical thickness results can be displayed on a brain surface, WM results must be displayed on the WM surface of the reference brain after its cortical mantle has been removed. This figure shows that reduced local volumes of brain tissue along the surface of the brain are distributed in perisylvian regions and they closely reflect underlying reductions in local volumes along the WM surface. AC, anterior cingulate; IFG, inferior frontal; LG, lingual gyrus; MFG, middle frontal gyrus; MTG, middle temporal gyrus; PreC, precentral gyrus; PC posterior cingulate; PoC, postcentral gyrus; SFG, superior frontal gyrus; SMG, supramarginal gyrus; STG, superior temporal gyrus.

**Figure 2:**
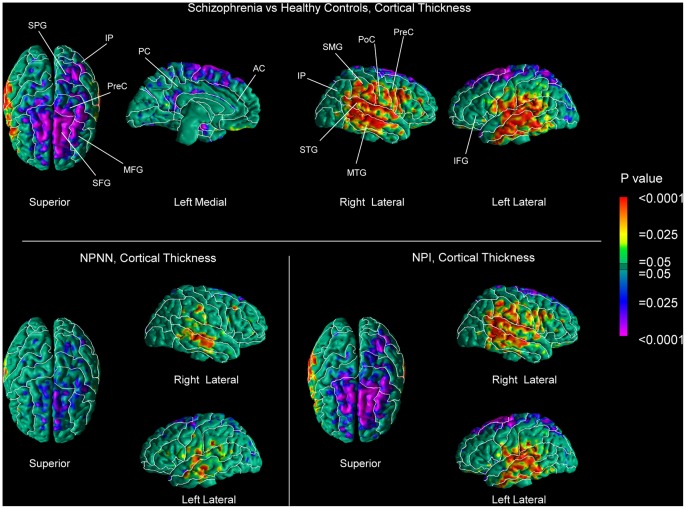
Maps of Group Differences in Cortical Thickness in Persons with Schizophrenia Compared with Healthy Controls. Statistical effects are color-encoded as in Fig. 1. Cortical thickness was rescaled for whole brain size. Results are GRF-corrected. Patients were divided in two subgroups: neuropsychologically near normal patients (NPNN; n = 21) (*Lower Left*) and neuropsychologically impaired (NPI; n = 50) (*Lower Right*) patients based on performance scores on the SPT. AC, anterior cingulate; IP, inferior parietal lobule; MFG, middle frontal gyrus; MTG, middle temporal gyrus; PC posterior cingulate; PoC, postcentral gyrus; PreC, precentral gyrus; SFG, superior frontal gyrus; SMG, supramarginal gyrus; SPG, superior parietal gyrus; STG, superior temporal gyrus. Other labels are the same as in Fig. 1. (*Upper*) Entire patient group compared with healthy controls. (*Lower Left*) NPNN subgroup (n = 21) compared with healthy controls (n = 57). (*Lower Right*) NPI subgroup (n = 50) compared with healthy controls. Patients exhibited greater cortical thickness in the perisylvian cortices in both hemispheres, as well as thinner cortices in the superior frontal, superior parietal and cingulate gyri. We did not observe any differences in the degree of cortical thickness when we compared neuropsychological groups directly to each other. These findings were preserved in unscaled data.

A comparison between patients and controls revealed extensive reductions of local volumes of brain tissue along the surface of the brain in an arc that included, laterally, the middle and inferior frontal gyri, most of the inferior half of the precentral and postcentral gyri, part of the supramarginal gyrus, and the superior and middle temporal gyri. Mesially, we detected reduced local volumes along the superior frontal, cingulate, and lingual gyri. We also observed reductions of local volumes of WM tissue along the underlying WM surface in a spatial pattern approximately corresponding to the perisylvian abnormalities we found along the surface of the brain (Fig. 1). Cortical thickness in the patient group followed a somewhat different pattern, with greater cortical thickness present in perisylvian cortices and thinner cortices in the superior and middle frontal gyri, left superior parietal lobule as well as along the cingulate gyrus (Fig. 2). The findings of greater cortical thickness were not affected when we did not rescale for brain size during the co-registration procedure.

### Neuropsychological Groups and Correlation with Working Memory Scores

**Figure 3:**
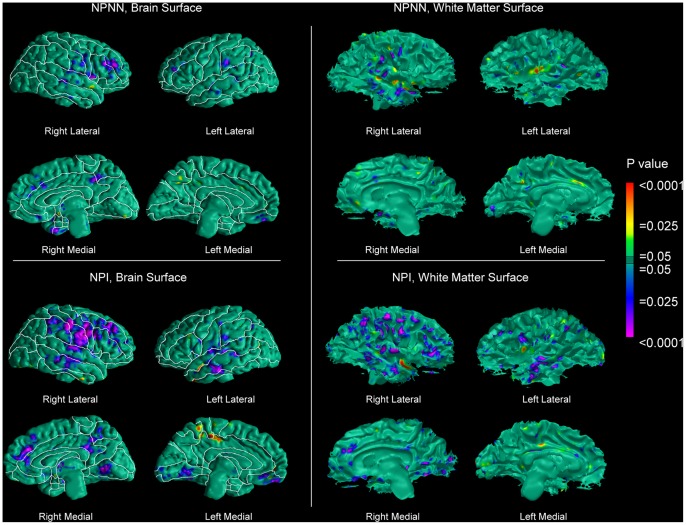
Maps of Group Differences in Local Volumes along the Surface of the Brain and of the Underlying WM Across Cognitive Subgroups. Statistical effects are color-encoded as in Fig. 1 and subgroup definitions are the same as in Fig. 2. Results are GRF-corrected. Reductions in local volumes of brain tissue along the surface of the brain and of the WM in perisylvian regions are more prominent and more extensive in the NPI group. (*Upper Left*) Comparisons of the brain surface of NPNN patients with that of healthy controls. (*Lower Left*) Comparisons of the brain surface of NPI patients with that of healthy controls. (*Upper Right*) Comparisons of the WM surface of NPNN patients with that of healthy controls. (*Lower Right*) Comparisons of the WM surface of NPI patients with that of healthy controls. Neuropsychologically impaired patients show a higher burden of anatomical abnormalities than neuropsychologically near-normal patients. Reductions of local volumes of brain tissue along the surface of the brain corresponded to reductions in local volumes along the surface of the underlying WM.

**Figure 4:**
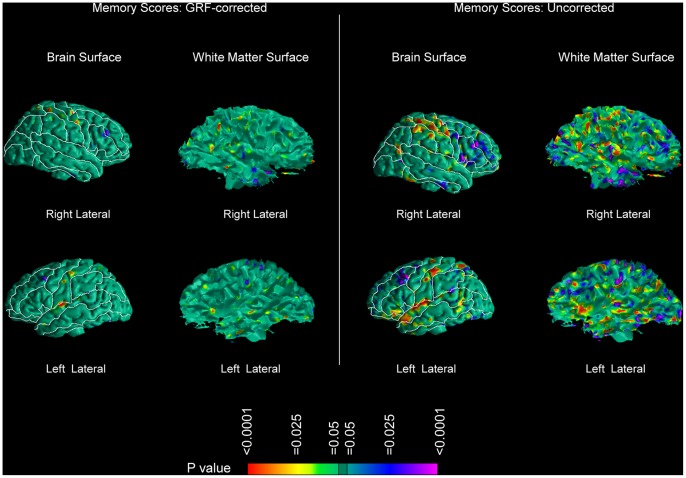
Correlations of Memory Scores with Local Volumes along the Surface of the Brain and of the Underlying WM. Statistical effects are color-encoded as in Fig. 1. (*Left Half of the Figure*) GRF-corrected results for the Brain Surface (Left Column) and for the WM Surface (Right Column). (*Right Half of the Figure*) Uncorrected results for the Brain Surface (Left Column) and for the WM Surface (Right Column). These figures demonstrate that increasing memory scores are associated with relatively greater local volumes along the WM surface. These correlations are within-group and they suggest that patients with better memory scores have a smaller degree of WM deficits. However, patients as a whole still have reductions in WM when compared with healthy controls, as shown in Fig. 1. Therefore the greater local volumes shown in this figure are not to be interpreted in the sense of an actual increase of WM in the patient group.

We observed reduced local volumes of brain tissue along the brain surface of NPI patients compared with that of controls, following a pattern similar to that of the entire patient sample, except in the superior temporal gyrus, where smaller abnormalities were present (Fig. 3). In contrast, the NPNN subgroup exhibited limited reductions in local volume along the brain surface of the middle frontal and posterior cingulate gyri (Fig. 3). In the NPI subgroup, the WM followed approximately the same spatial pattern, with reduced local volumes of WM along the surface of the WM of the right hemisphere. We found only minimal abnormalities in the WM of the NPNN subgroup (Fig. 3). Both subgroups compared with controls exhibited some degree of greater cortical thickness in perisylvian cortices and of reduced thickness in the superior frontal gyri, in a pattern seemingly more pronounced in the NPI group (Fig. 2). However, we did not detect statistically significant differences in cortical thickness between the NPI and the NPNN subgroups when we compared them directly to one another. Uncorrected comparison of the WM surface between the NPI and NPNN patients revealed reduced local WM volumes along the WM surface underlying the perisylvian cortices in the patients with impaired memory performances. GRF-corrected analyses pointed in the same direction, showing small volumetric reductions along the WM surface in the NPI subgroup (Fig. S1). Further, analyses of the WM surface showed that overall memory scores, as measured on the SPT, were associated with greater local WM volumes along the WM surface, especially in the right hemisphere in areas underlying temporal, parietal, and sensorimotor cortices. These local volumes were greater in a relative sense, given that the WM was still reduced in patients compared to controls, though relatively less so in patients with higher memory scores (Fig. 4). We did not detect any correlations of cortical thickness with memory scores.

### Correlation with Symptom Severity

**Figure 5:**
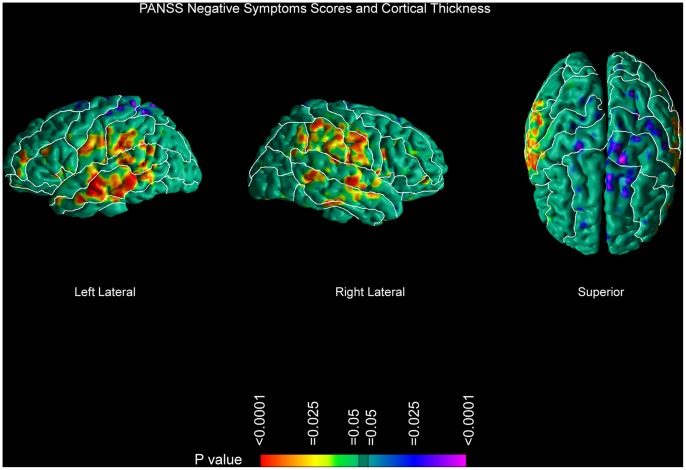
Correlations of Cortical Thickness with Severity of Negative Symptoms. Statistical effects are color-encoded as in Fig. 1. This figure shows correlations of cortical thickness measures with negative symptoms scores from the PANSS. All correlations are GRF-corrected. Increased symptom burden was associated with greater thickness in perisylvian cortices but only minimal thinning in the left superior and left precentral gyrus (compare with Fig. 2). We did not find any association of WM surface morphology with symptom scores.

Only negative symptoms clearly correlated with measures of cortical thickness, with increasing symptom burden accompanying relative greater thickness of the perisylvian cortices bilaterally. We found no significant correlation of symptom severity with measures of local volume along the surface of the brain and of the WM (Fig. 5).

### Scaling Effects

Results for analyses of the brain surface performed without scaling correction are reported in Fig. S2.

### Total Surface Area

In the right hemisphere, patients showed smaller WM surface area than controls (triangulation method: p = 0.016; voxel method: p = 0.004), after controlling for all other covariates. When we estimated the main effect of gender, regardless of diagnostic group membership, we found that males showed, in the right hemisphere, a larger WM surface area than females (both methods: p = 0.001). Regression of the entire eroded cortical surface area (left and right hemispheres combined) onto age, age^2^, gender and diagnostic group showed that patients had smaller cortical surface area than healthy controls (triangulation method: p = 0.028; voxel method: p = 0.05).

## Discussion

Abnormalities of local volumes of brain tissue along the surface of the brain were associated with abnormalities of local volumes of WM tissue along the surface of the underlying WM at corresponding locations, rather than with reduced thickness of cortical GM. Reductions of local volumes along the brain surface did not co-localize with areas of thinner cortex, as we observed greater cortical thickness in those locations instead. Whether we controlled or not for the effects of cortical thickness on morphology of the brain surface, our results did not change, quantitatively supporting our conclusion that the abnormalities along the brain surface do not reflect abnormalities of the underlying cortical thickness, but rather of the underlying white matter. Additionally, we observed smaller total surface areas of the cortex and of the WM, leading us to conclude that the regional GM volume reductions we had previously reported are partially attributable to reduced cortical surface area, accompanied by an underlying reduction in WM surface area.

These findings converge with those from other studies suggesting that WM may be an important contributor to the anatomical disturbances observed in schizophrenia. The WM abnormalities were located primarily in the region of the arcuate fasciculus. Reduced local WM volumes were also present along the WM surface of the anterior and posterior cingulate gyri. The locations of these findings replicate the locations of abnormalities in cortical GM volume reported in prior studies, in both first episode and chronic patients, particularly in fronto-temporal, inferior sensorimotor, inferior parietal, cingulate, and lingual cortices. The specific location of WM abnormalities within perisylvian and limbic areas is not unexpected, as these are regions thought to underlie auditory hallucinations and formal thought disorder, impaired language functioning, and the emotional disturbances observed in schizophrenia. WM findings were most prominent in the right hemisphere, consistent with a prior longitudinal study that reported a lag in WM growth greatest in the right hemispheres of persons with childhood-onset schizophrenia [38]. The validity of the WM abnormalities is furthermore indirectly supported by postmortem studies reporting myelin and oligodendrocyte disturbances in schizophrenia.

Partially contradicting our expectations, we detected thicker perisylvian cortices in patients. We did find, however, thinner cortex in the superior and middle frontal gyri, the superior parietal lobule, and along the cingulate gyrus, consistent with prior findings of reduced cortical thickness observed in these regions. Among the studies that have measured cortical thickness directly, most have detected thinning [39] in frontal, temporal, and cingulate cortices. We speculate that the thicker cortices we found in patients could be associated with medication use or any other illness-related factor such as hospitalization or chronicity but these explanations cannot be proved, given that virtually all patients in this sample were on medications and they were imaged at only a single point in time.

### Correlations with Measures of Cognitive Functioning

Poorer performance on working memory tasks accompanied more extensive abnormalities of local volumes along the surfaces of brain and of the underlying WM. We previously demonstrated in this same sample that NPI and NPNN patients differ in regional anatomical volumes, with the NPI group showing reduced regional GM and WM volumes and the NPNN group exhibiting only abnormal regional volumes of GM [18]. Our results confirm and extend these observations by indicating that those who are neuropsychologically impaired carry a more extensive burden of local anatomical abnormalities deriving from white matter. Moreover, individual correlations with overall memory scores seemed to support this interpretation, as greater impairments in working memory are associated with a greater degree of WM compromise (Fig. 4). The association of WM with measures of neuropsychological performance is consistent with evidence from imaging genetics studies [40], as well as from recent-onset schizophrenia studies [41] and WM loss has a stronger association with cognitive function than GM does [42]. WM deficits in locations similar to ours and associated with a variety of cognitive measures have been observed in samples of minimally medicated patients at the onset of illness, suggesting that these findings are not due to illness chronicity or medication exposure [43].

### Correlations with Symptom Severity

Severity of negative symptoms correlated with greater cortical thickness in perisylvian cortices and not with deficits in WM as we had hypothesized. The interpretation of these findings is unclear, given the absence of significant correlations with measures of local volume along the surfaces of the brain and of the underlying WM in corresponding locations. We cannot determine whether these abnormalities in thickness are primary or secondary to compensatory changes or medication use. In general, however, associations of anatomical measures with symptom dimensions tend to be weak [42].

Taken together, our results converge with those of prior studies to suggest that an important anatomical abnormality in schizophrenia involves the WM that connects together important cortical association areas [44]. Structural connectivity [45] and functional network analyses [46] have supported the hypothesis that schizophrenia may be a disconnection syndrome based within WM. Rather than suggesting diffuse WM damage, the perisylvian location of our WM findings points to circuit-based WM abnormalities, disrupting the WM that connects frontal, parietal and temporal regions, consistent with prior reports of WM abnormalities in frontal cortex, internal capsule [2], fornix and cingulum [47], and arcuate fasciculus [48]. We detected a reduction of total cortical surface area and WM surface area in the schizophrenia group, generally consistent with previous observations [49]. A reduced total cortical surface area may contribute, along with reduced cortical thickness, to the smaller GM volumes previously reported in our patient sample [18], although the global nature of the measurement did not allow determination of the regional localization of abnormalities in cortical surface area.

Recently, abnormalities in both cortical surface area and WM anatomy [2] have been proposed as strong candidates for markers of trait vulnerability in schizophrenia. Indeed, imaging studies in monozygotic and dizygotic twins discordant for schizophrenia suggest that WM disturbances are a marker of genetic vulnerability for this disease [50], whereas cortical thickness abnormalities are most likely secondary to a separate underlying disease process. Consistent with this proposed state-related effect for abnormalities in cortical thickness, a large recent study failed to detect significant reductions in cortical thickness in unaffected siblings compared with persons affected with schizophrenia and healthy controls, again suggesting that cortical thinning is unlikely to be a state-independent marker for this illness [51].

Additionally, findings from anatomical studies in cohorts at high-risk for psychosis appear to agree with our observations. For instance, the location of the cortical thinning we observed is consistent with findings from prodromal cohorts showing possible GM loss in prefrontal cortices [52]. Similarly, disruptions of WM integrity and development have been observed before the onset of psychosis in areas underlying or connecting association cortices [53]–[56]. Despite these similarities, however, anatomical studies in high-risk cohorts are few in number, and the high risk and prodromal samples differ greatly from ours, making comparison of our findings difficult. Only longitudinal studies of prodromal patients followed longitudinally through the chronic phase would permit us to know how anatomical abnormalities in the prodrome compare with those observed in the chronic phase of schizophrenic illness.

In conclusion, we speculate that GM and WM volumes are both reduced in adults suffering from schizophrenia, but the various morphological components of these volumetric abnormalities may be under different pathogenic influences [57]. Independent replication with studies using different samples and different analytic approaches are ultimately needed to confirm our results.

### Limitations

The findings of this study should be viewed in light of several limitations. 1) Imaging analyses, in general, do not permit identification of the underlying histological determinants of the observed anatomical abnormalities. 2) Although WM seems to account for our volumetric findings on the cerebral surface in schizophrenia, findings from several postmortem studies suggest that histological abnormalities are present in cortical gray matter. 3).

Prior imaging studies have implicated the cerebellum and the insula in the pathogenesis of schizophrenia. The cerebellum, however, was not included in our analyses. We were also unable to assess the morphological features of the insula, because this structure is not visible on the cortical surface. This is an important limitation, given recent observations of significantly pronounced hypogyria in the left insula of patients with schizophrenia [58]. 4) The correlation of PANSS scores with measures of surface morphology should be interpreted with caution because scores were not available for all patients. 5) Neuropsychological measures were not available from domains other than working memory, verbal memory, and attention.

An important limitation of our study is that our sample comprised only medicated and chronically ill patients. Therefore, similar to many prior studies of already-affected patients, we could not entirely disentangle the effects of individual medication classes from disease effects on morphological measures. Assessing which medications each patient has been exposed to is notoriously difficult in this population, more so the longer the illness has been present. Poor compliance and poor recall are frequent. Such patients have had multiple hospitalizations, at times in different geographical locations, with difficulty assembling complete records that span several years, if not decades. Furthermore, higher doses of antipsychotic medication tend to correlate with severity of illness, and therefore illness and medication doses are inextricably entangled. Additionally, the determination of chlorpromazine equivalents has historically been based on the assessment of clinical effects, providing no information on whether these medications have equivalent biological effects on brain structure. Moreover, the determination of these equivalents relies on placebo-controlled fixed-dose studies that establish minimum clinically effective doses. Thus, chlorpromazine equivalency determined at one point of the dosage range for a particular medication does not imply that the same equivalency holds at higher doses for that same medication [59]. To complicate matters, although chlorpromazine equivalents frequently are included as statistical covariates, this approach assumes that the effects of these medications on anatomical variables are linear and that the same statistical relationship is valid across the entire brain. For these reasons, the scalar product we used as a proxy for cumulative-life exposure can only be a very rough approximation. Therefore, medication effects cannot be truly assessed in a chronic population via statistical modeling. First-episode studies, animal studies, and prodromal studies are more suited to answer these questions. Nevertheless, we know medications affect anatomical measures, as it had been previously demonstrated by post-mortem studies in primates [60] and proposed by imaging studies [37], [61]. For this reason, despite all the limitations of statistical covariation, we did attempt to control for cumulative exposure to antipsychotic medication in a manner similar to previous studies [37], finding no evidence for an effect either on cortical thickness or on local volumes along the surfaces of the cortical mantle and of the underlying WM.

Although our sample of patients and healthy controls were matched for age, matching for minority status was not possible. The patient group had a borderline higher proportion of males, common in samples of patients with chronic schizophrenia, with many studies even including only male participants [45]. We assessed the effects of both these variables on all anatomical measures (Figs. S3, S5) and controlled for them when appropriate. Finally, this dataset was analyzed in a prior paper, theoretically introducing the possibility of type-I error. Nevertheless, the imaging techniques used in this paper differ dramatically from the conventional ROI-based measures of volume reported previously, allowing a finer-grained analysis of brain structure.

## Supporting Information

Figure S1 **Direct Statistical Comparison of Local Volumes along the Surface of the WM between NPI and NPNN Patients after Covarying for Age, Age^2^ and Gender.** Direct statistical comparisons are depicted between patients with impaired neuropsychological performance (NPI) and patients with near normal neuropsychological performance (NPNN). Statistical effects are color-encoded as in Fig. 1. The figure demonstrates that the NPI subgroup has reduced local volumes of WM along the surface of the WM when compared to NPNN subgroup. The findings survive correction for multiple comparisons. (*Upper*) Results after GRF- correction. (*Lower*) Uncorrected results.
(TIF)

Figure S2 **Comparison of Local Volumes along the Surface of the Brain between Persons with Schizophrenia and Healthy Controls without Scaling Correction.** Statistical effects are color -encoded as in Fig. 1. Results are GRF-corrected and should be compared with the scaled results for the same comparison in Fig. 1. Briefly, when analyzing unscaled data, we observed, in the patient group, generalized reductions in local volumes of brain tissue along the entire medial and lateral brain surfaces, more prominent in the right than in the left hemisphere. This figure shows that when we do not correct for whole brain volume, patients with schizophrenia exhibit overall smaller cerebral volume than healthy controls. The comparison between scaled and unscaled cortical thickness data did not detect significant differences (not shown), likely because the scaling relationship of brain surface with overall brain size was greater than that of cortical thickness with overall brain size.
(TIF)

Figure S3 **Main Effects of Gender on Cortical Thickness** ***(Top)*** **and on Local Volumes along the Surface of the WM** ***(Bottom)*** **in Patients with Schizophrenia.** Statistical effects are color -encoded as in Fig. 1. Results are GRF-corrected. This figure shows greater local volumes along the surface of the WM in male patients relative to female patients, mostly in the superior frontal gyrus. Males also show thinner cortices in the same location compared with females. These results are consistent with our quantitative finding that male patients exhibit larger total surface area of the WM than female patients. A similar pattern has been previously observed both in patients and in healthy control samples. We do not report effects in our healthy control group, which consisted mainly of small changes in local volumes along the surface of the WM in the left hemisphere, because these findings were much less significant and therefore of difficult interpretation.
(TIF)

Figure S4 **Age by Diagnosis Interactions. Local Volumes along the Surface of the WM of the Right Hemisphere (** ***Top*** **) and of the Left Hemisphere (** ***Bottom*** **).** Statistical effects are color -encoded as in Fig. 1. Results are GRF-corrected. This figure shows greater local volumes along the surface of the WM of the anterior cingulum (underlying the ACC) associated with age, as well as correspondingly greater local volumes along the surface of the brain. No interaction effects on cortical thickness were observed. The interaction term captures the different relationship of WM with age in the healthy controls and in the patient group. This interaction term is significant in voxels different than those where the main effects of diagnosis were observed, and it is highly significant in the cingulum just anterior to the genu of the corpus callosum. Plotting the relationship between age and anatomical measures (not reported) allowed us to conclude that this interaction reflected greater local WM volumes along the surface of the WM of the anterior cingulum in progressively older patients. Conversely, healthy controls exhibited progressively smaller local volumes with increasing age in this same region. This increase notwithstanding, when patients are compared to healthy controls, they do show diffuse reductions in WM (Fig. 1). We do not report the main effects for age and age^2^ because they were minimal and thus of difficult interpretation. Briefly, they consisted in a very small increase in local volumes at the WM surface of the superior frontal gyrus as well as a decrease in the same area associated with its quadratic term (age^2^). The combination of these effects followed an inverted U curve. This pattern was identical at the brain surface. No abnormalities in cortical thickness were observed.
(TIF)

Figure S5 **Main Effects of Minority Status on Cortical Thickness in Patients with Schizophrenia.** Statistical effects are color encoded as in Fig. 1. Results are GRF-corrected. Region definition was not applied. This figure shows correlations of measures of cortical thickness with minority status. Minority status was defined for the purposes of this analysis as being non-Caucasian. Minority patients exhibit thicker cortices in the superior and middle frontal gyri as well as along the cingulate gyri. Although the effect of minority group position has already been reported in psychotic syndromes, the specific meaning of increased cortical thickness in our sample is unclear, but perhaps related to possible differential environmental exposures. Main effects for minority status were not observed in the healthy control group.
(TIF)

Methods S1 **Supplementary Methods.**
(DOC)

## References

[1] Yamasue H, Iwanami A, Hirayasu Y, Yamada H, Abe O, et al (2004) Localized volume reduction in prefrontal, temporolimbic, and paralimbic regions in schizophrenia: an MRI parcellation study. Psychiatry Res. 131: 195–207. 10.1016/j.pscychresns.2004.05.004 15465289

[2] Di X, Chan RC, Gong QY (2009) White matter reduction in patients with schizophrenia as revealed by voxel-based morphometry: an activation likelihood estimation meta-analysis. Prog Neuropsychopharmacol Biol Psychiatry. 33: 1390–1394. 10.1016/j.pnpbp.2009.08.020 19744536

[3] Honea R, Crow TJ, Passingham D, Mackay CE (2005) Regional deficits in brain volume in schizophrenia: a meta-analysis of voxel-based morphometry studies. Am J Psychiatry. 162: 2233–2245. 10.1176/appi.ajp.162.12.2233 16330585

[4] Bansal R, Staib LH, Whiteman R, Wang YM, Peterson BS (2005) ROC-based assessments of 3D cortical surface-matching algorithms. Neuroimage. 24: 150–162. 10.1016/j.neuroimage.2004.08.054 15588606

[5] Schultz CC, Koch K, Wagner G, Roebel M, Schachtzabel C, et al (2010) Reduced cortical thickness in first episode schizophrenia. Schizophr Res. 116: 204–209. 10.1016/j.schres.2009.11.001 19926451

[6] Sun D, Stuart GW, Jenkinson M, Wood SJ, McGorry PD, et al (2009) Brain surface contraction mapped in first-episode schizophrenia: a longitudinal magnetic resonance imaging study. Mol Psychiatry. 14: 976–986. 10.1038/mp.2008.34 PMC2773126 18607377

[7] Palaniyappan L, Mallikarjun P, Joseph V, White TP, Liddle PF (2011) Folding of the prefrontal cortex in schizophrenia: regional differences in gyrification. Biol Psychiatry. 69: 974–979. 10.1016/j.biopsych.2010.12.012 21257157

[8] Winkler AM, Kochunov P, Blangero J, Almasy L, Zilles K, et al (2010) Cortical thickness or grey matter volume? The importance of selecting the phenotype for imaging genetics studies. Neuroimage. 53: 1135–1146. 10.1016/j.neuroimage.2009.12.028 PMC2891595 20006715

[9] Panizzon MS, Fennema-Notestine C, Eyler LT, Jernigan TL, Prom-Wormley, et al (2009) Distinct genetic influences on cortical surface area and cortical thickness. Cereb Cortex. 19: 2728–2735. 10.1093/cercor/bhp026 PMC2758684 19299253

[10] Rakic P (1988) Specification of cerebral cortical areas. Science. 241: 170–176. 10.1126/science.3291116 3291116

[11] Huttenlocher PR, Dabholkar AS (1997) Regional differences in synaptogenesis in human cerebral cortex. J Comp Neurol. 387: 167–178. 10.1002/(sici)1096-9861(19971020)387:2<167::aid-cne1>3.0.co;2-z 9336221

[12] Landing BH, Shankle WR, Hara J, Brannock J, Fallon JH (2002) The development of structure and function in the postnatal human cerebral cortex from birth to 72 months: changes in thickness of layers II and III co-relate to the onset of new age-specific behaviors. Pediatr Pathol Mol Med. 21: 321–342. 10.1080/02770930290056541 12056506

[13] Paus T, Keshavan M, Giedd JN (2008) Why do many psychiatric disorders emerge during adolescence? Nat Rev Neurosci. 9: 947–957. 10.1038/nrn2513 PMC2762785 19002191

[14] Petanjek Z, Judas M, Simic G, Rasin MR, Uylings HB, et al (2011) Extraordinary neoteny of synaptic spines in the human prefrontal cortex. Proc Natl Acad Sci U S A. 108: 13281–13286. 10.1073/pnas.1105108108 PMC3156171 21788513

[15] Faludi G, Mirnics K (2011) Synaptic changes in the brain of subjects with schizophrenia. Int J Dev Neurosci. 29: 305–309. 10.1016/j.ijdevneu.2011.02.013 PMC3074034 21382468

[16] Boksa P (2012) Abnormal synaptic pruning in schizophrenia: Urban myth or reality? J Psychiatry Neurosci. 37: 75–77. 10.1503/jpn.120007 PMC3297065 22339991

[17] Bansal R, Staib LH, Xu D, Zhu H, Peterson BS (2007) Statistical analyses of brain surfaces using Gaussian random fields on 2-D manifolds. IEEE Trans Med Imaging. 26: 46–57. 10.1109/TMI.2006.884187 PMC2366175 17243583

[18] Wexler BE, Zhu H, Bell MD, Nicholls SS, Fulbright RK, et al (2009) Neuropsychological Near Normality and Brain Structure Abnormality in Schizophrenia. Am J Psychiatry 166: 189–195. 10.1176/appi.ajp.2008.08020258 PMC4288572 18765481

[19] Whitford TJ, Grieve SM, Farrow TF, Gomes L, Brennan J, et al (2007) Volumetric white matter abnormalities in first-episode schizophrenia: a longitudinal, tensor-based morphometry study. Am J Psychiatry. 164: 1082–1089. 10.1176/ajp.2007.164.7.1082 17606660

[20] Palaniyappan L, Liddle PF (2012) Differential effects of surface area, gyrification and cortical thickness on voxel based morphometric deficits in schizophrenia. Neuroimage. 60: 693–699. 10.1016/j.neuroimage.2011.12.058 22227049

[21] Karlsgodt KH, van Erp TG, Poldrack RA, Bearden CE, Nuechterlein KH, et al (2008) Diffusion tensor imaging of the superior longitudinal fasciculus and working memory in recent-onset schizophrenia. Biol Psychiatry. 63: 512–518. 10.1016/j.biopsych.2007.06.017 17720147

[22] Nesvag R, Saetre P, Lawyer G, Jonsson EG, Agartz I (2009) The relationship between symptom severity and regional cortical and grey matter volumes in schizophrenia. Prog Neuropsychopharmacol Biol Psychiatry. 33: 482–490. 10.1016/j.pnpbp.2009.01.013 19439246

[23] Makris N, Seidman LJ, Ahern T, Kennedy DN, Caviness VS, et al (2010) White matter volume abnormalities and associations with symptomatology in schizophrenia. Psychiatry Res. 183: 21–29. 10.1016/j.pscychresns.2010.04.016 PMC2913317 20538438

[24] Stephan KE, Friston KJ, Frith CD (2009) Dysconnection in schizophrenia: from abnormal synaptic plasticity to failures of self-monitoring. Schizophr Bull. 35: 509–527. 10.1093/schbul/sbn176 PMC2669579 19155345

[25] Ellison-Wright I, Bullmore E (2009) Meta-analysis of diffusion tensor imaging studies in schizophrenia. Schizophr Res. 108: 3–10. 10.1016/j.schres.2008.11.021 19128945

[26] Burns J, Job D, Bastin ME, Whalley H, Macgillivray T, et al (2003) Structural disconnectivity in schizophrenia: a diffusion tensor magnetic resonance imaging study. Br J Psychiatry. 182: 439–443. 12724248

[27] McGuire PK, Frith CD (1996) Disordered functional connectivity in schizophrenia. Psychol Med. 26: 663–667. 10.1017/s0033291700037673 8817700

[28] Friston KJ (1998) The disconnection hypothesis. Schizophr Res. 30: 115–125. 10.1016/s0920-9964(97)00140-0 9549774

[29] Saykin AJ, Shtasel DL, Gur RE, Kester DB, Mozley LH, et al (1994) Neuropsychological deficits in neuroleptic naive patients with first-episode schizophrenia. Arch Gen Psychiatry. 51: 124–131. 10.1001/archpsyc.1994.03950020048005 7905258

[30] Delis D, Kramer J, Kaplan E, & Ober B (1987) California Verbal Learning Test. San Antonio, TX: The Psychological Corporation.

[31] Kay SR, Opler LA, Lindenmayer JP (1989) The Positive and Negative Syndrome Scale (PANSS): rationale and standardisation. Br J Psychiatry Suppl.59–67. 2619982

[32] Sled JG, Zijdenbos AP, Evans AC (1998) A nonparametric method for automatic correction of intensity nonuniformity in MRI data. IEEE Trans Med Imaging. 17: 87–97. 10.1109/42.668698 9617910

[33] Shrout PE, Fleiss JL (1979) Intraclass correlations: uses in assessing rater reliability. Psychol Bull. 86: 420–428. 10.1037//0033-2909.86.2.420 18839484

[34] Mazziotta J, Toga AW, Evans A, Fox P, Lancaster J, et al (2001) A probabilistic atlas and reference system for the human brain: International Consortium for Brain Mapping (ICBM). Philos Trans R Soc London B Biol Sci. 356: 1293–1322. 10.1098/rstb.2001.0915 PMC1088516 11545704

[35] Rosenfeld A, Pfaltz JL (1968) Distance Functions in Digital Pictures. Pattern Recognition. 1: 33–61.

[36] Prasad KM, Goradia D, Eack S, Rajagopalan M, Nutche J, et al (2010) Cortical surface characteristics among offspring of schizophrenia subjects. Schizophr Res. 116: 143–151. 10.1016/j.schres.2009.11.003 PMC2818600 19962858

[37] Nesvag R, Lawyer G, Varnas K, Fjell AM, Walhovd KB, et al (2008) Regional thinning of the cerebral cortex in schizophrenia: effects of diagnosis, age and antipsychotic medication. Schizophr Res. 98: 16–28. 10.1016/j.schres.2007.09.015 17933495

[38] Gogtay N, Lu A, Leow AD, Klunder AD, Lee AD, et al (2008) Three-dimensional brain growth abnormalities in childhood-onset schizophrenia visualized by using tensor-based morphometry. Proc Natl Acad Sci U S A. 105: 15979–15984. 10.1073/pnas.0806485105 PMC2566993 18852461

[39] Narr KL, Bilder RM, Toga AW, Woods RP, Rex DE, et al (2005) Mapping cortical thickness and gray matter concentration in first episode schizophrenia. Cereb Cortex. 15: 708–719. 10.1093/cercor/bhh172 15371291

[40] Zuliani R, Moorhead TW, Bastin ME, Johnstone EC, Lawrie SM, et al (2011) Genetic variants in the ErbB4 gene are associated with white matter integrity. Psychiatry Res. 191: 133–137. 10.1016/j.pscychresns.2010.11.001 PMC5372351 21232925

[41] Szeszko PR, Robinson DG, Ashtari M, Vogel J, Betensky J, et al (2008) Clinical and neuropsychological correlates of white matter abnormalities in recent onset schizophrenia. Neuropsychopharmacology. 33: 976–984. 10.1038/sj.npp.1301480 17581532

[42] Andreasen NC, Nopoulos P, Magnotta V, Pierson R, Ziebell S, et al (2011) Progressive Brain Change in Schizophrenia: A Prospective Longitudinal Study of First-Episode Schizophrenia. Biol Psychiatry. 70: 672–679. 10.1016/j.biopsych.2011.05.017 PMC3496792 21784414

[43] Perez-Iglesias R, Tordesillas-Gutierrez D, McGuire PK, Barker GJ, Roiz-Santianez R, et al (2010) White matter integrity and cognitive impairment in first-episode psychosis. Am J Psychiatry. 167: 451–458. 10.1176/appi.ajp.2009.09050716 20160006

[44] Phillips OR, Nuechterlein KH, Asarnow RF, Clark KA, Cabeen R, et al (2011) Mapping Corticocortical Structural Integrity in Schizophrenia and Effects of Genetic Liability. Biol Psychiatry. 70: 680–689. 10.1016/j.biopsych.2011.03.039 PMC3838300 21571255

[45] Corradi-Dell’acqua C, Tomelleri L, Bellani M, Rambaldelli G, Cerini R, et al (2011) Thalamic-insular dysconnectivity in schizophrenia: Evidence from structural equation modeling. Hum Brain Mapp. 33: 740–752. 10.1002/hbm.21246 PMC6870155 21484952

[46] Lynall ME, Bassett DS, Kerwin R, McKenna PJ, Kitzbichler M, et al (2010) Functional connectivity and brain networks in schizophrenia. J Neurosci. 30: 9477–9487. 10.1523/JNEUROSCI.0333-10.2010 PMC2914251 20631176

[47] Abdul-Rahman MF, Qiu A, Sim K (2011) Regionally specific white matter disruptions of fornix and cingulum in schizophrenia. PLoS One. 6: e18652. 10.1371/journal.pone.0018652 PMC3077390 21533181

[48] Catani M, Craig MC, Forkel SJ, Kanaan R, Picchioni M, et al (2011) Altered integrity of perisylvian language pathways in schizophrenia: relationship to auditory hallucinations. Biol Psychiatry. 70: 1143–1150. 10.1016/j.biopsych.2011.06.013 21798516

[49] Palaniyappan L, Mallikarjun P, Joseph V, White TP, Liddle PF (2011) Regional contraction of brain surface area involves three large-scale networks in schizophrenia. Schizophr Res. 129: 163–168. 10.1016/j.schres.2011.03.020 21497489

[50] Hulshoff Pol HE, Brans RG, van Haren NE, Schnack HG, Langen M, et al (2004) Gray and white matter volume abnormalities in monozygotic and same-gender dizygotic twins discordant for schizophrenia. Biol Psychiatry. 55: 126–130. 10.1016/s0006-3223(03)00728-5 14732591

[51] Goldman AL, Pezawas L, Mattay VS, Fischl B, Verchinski BA, et al (2009) Widespread reductions of cortical thickness in schizophrenia and spectrum disorders and evidence of heritability. Arch Gen Psychiatry. 66: 467–477. 10.1001/archgenpsychiatry.2009.24 PMC2719488 19414706

[52] Sun D, Phillips L, Velakoulis D, Yung A, McGorry PD, et al (2009) Progressive brain structural changes mapped as psychosis develops in ‘at risk’ individuals. Schizophr Res. 108: 85–92. 10.1016/j.schres.2008.11.026 PMC2670732 19138834

[53] Ziermans TB, Schothorst PF, Schnack HG, Koolschijn PC, Kahn RS, et al (2012) Progressive structural brain changes during development of psychosis. Schizophr Bull. 38: 519–530. 10.1093/schbul/sbq113 PMC3329986 20929968

[54] Smieskova R, Fusar-Poli P, Allen P, Bendfeldt K, Stieglitz RD, et al (2010) Neuroimaging predictors of transition to psychosis–a systematic review and meta-analysis. Neurosci Biobehav Rev. 34: 1207–1222. 10.1016/j.neubiorev.2010.01.016 20144653

[55] Fusar-Poli P, Borgwardt S, Bechdolf A, Addington J, Riecher-Rossler A, et al (2012) The Psychosis High-Risk State: A Comprehensive State-of-the-Art Review. Arch Gen Psychiatry 19: 1–14. 10.1001/jamapsychiatry.2013.269 PMC4356506 23165428

[56] Carletti F, Woolley JB, Bhattacharyya S, Perez-Iglesias R, Fusar Poli P, et al (2012) Alterations in white matter evident before the onset of psychosis. Schizophr Bull. 38: 1170–1179. 10.1093/schbul/sbs053 PMC3494044 22472474

[57] Haijma SV, Van Haren N, Cahn W, Koolschijn PC, Hulshoff Pol HE, et al.. (2012) Brain Volumes in Schizophrenia: A Meta-Analysis in Over 18 000 Subjects. Schizophr Bull.Epub 10.1093/schbul/sbs118 PMC3756785 23042112

[58] Palaniyappan L, Liddle PF (2012) Aberrant cortical gyrification in schizophrenia: a surface-based morphometry study. J Psychiatry Neurosci. 37: 399–406. 10.1503/jpn.110119 PMC3493098 22640702

[59] Woods SW (2003) Chlorpromazine equivalent doses for the newer atypical antipsychotics. J Clin Psychiatry. 64: 663–667. 10.4088/jcp.v64n0607 12823080

[60] Konopaske GT, Dorph-Petersen KA, Pierri JN, Wu Q, Sampson AR, et al (2007) Effect of chronic exposure to antipsychotic medication on cell numbers in the parietal cortex of macaque monkeys. Neuropsychopharmacology. 32: 1216–1223. 10.1038/sj.npp.1301233 17063154

[61] Ho BC, Andreasen NC, Ziebell S, Pierson R, Magnotta V (2011) Long-term antipsychotic treatment and brain volumes: a longitudinal study of first-episode schizophrenia. Arch Gen Psychiatry. 68: 128–137. 10.1001/archgenpsychiatry.2010.199 PMC3476840 21300943

