# Correction: Anatomical Abnormalities in Gray and White Matter of the Cortical Surface in Persons with Schizophrenia

**DOI:** 10.1371/annotation/52ec57b7-e60d-40a8-b4fb-9bce5f9f9b40

**Published:** 2013-11-12

**Authors:** Tiziano Colibazzi, Bruce E. Wexler, Ravi Bansal, Xuejun Hao, Jun Liu, Juan Sanchez-Peña, Cheryl Corcoran, Jeffrey A. Lieberman, Bradley S. Peterson

Several errors were introduced in the preparation of this article for publication:

1.) In the methods section under Statistical Analysis the formulas should show i is a subscript. Please see the correct equations here: 

**Figure:**
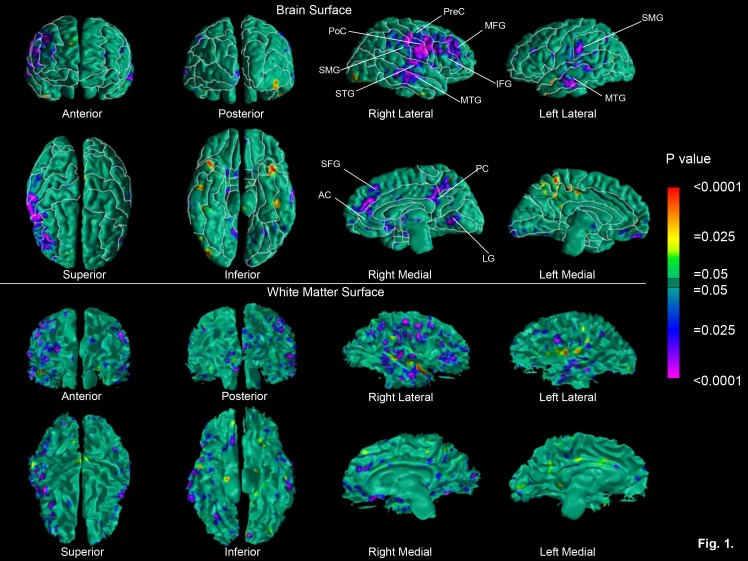


Please see the correction Figure 2 here: 

**Figure:**
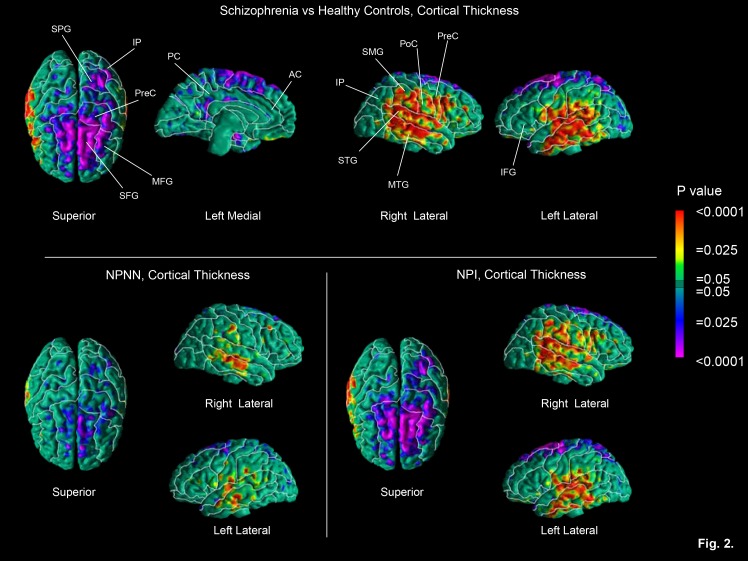


Please see the correction Figure 3 here: 

**Figure:**
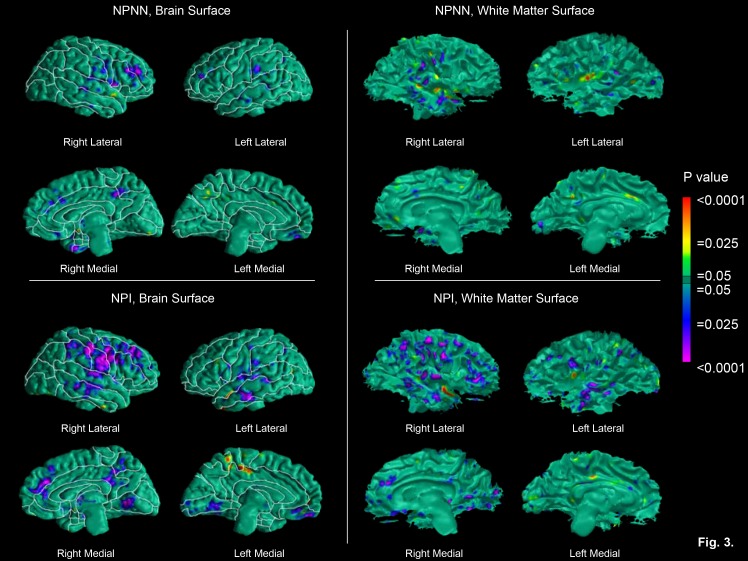


3.) In the discussion section, the second paragraph under limitations should be included in the first paragraph.

Here is the correct paragraph: The findings of this study should be viewed in light of several limitations. 1) Imaging analyses, in general, do not permit identification of the underlying histological determinants of the observed anatomical abnormalities. 2) Although WM seems to account for our volumetric findings on the cerebral surface in schizophrenia, findings from several postmortem studies suggest that histological abnormalities are present in cortical gray matter. 3). Prior imaging studies have implicated the cerebellum and the insula in the pathogenesis of schizophrenia. The cerebellum, however, was not included in our analyses. We were also unable to assess the morphological features of the insula, because this structure is not visible on the cortical surface. This is an important limitation, given recent observations of significantly pronounced hypogyria in the left insula of patients with schizophrenia [58]. 4) The correlation of PANSS scores with measures of surface morphology should be interpreted with caution because scores were not available for all patients. 5) Neuropsychological measures were not available from domains other than working memory, verbal memory, and attention. 

