# Supplementary figures and images for: Anatomical Abnormalities in Gray and White Matter of the Cortical Surface in Persons with Schizophrenia
Source: PLoS One. 2013 Feb 13;8(2):e55783. doi: 10.1371/journal.pone.0055783 (PMC3572102; doi:10.1371/journal.pone.0055783)

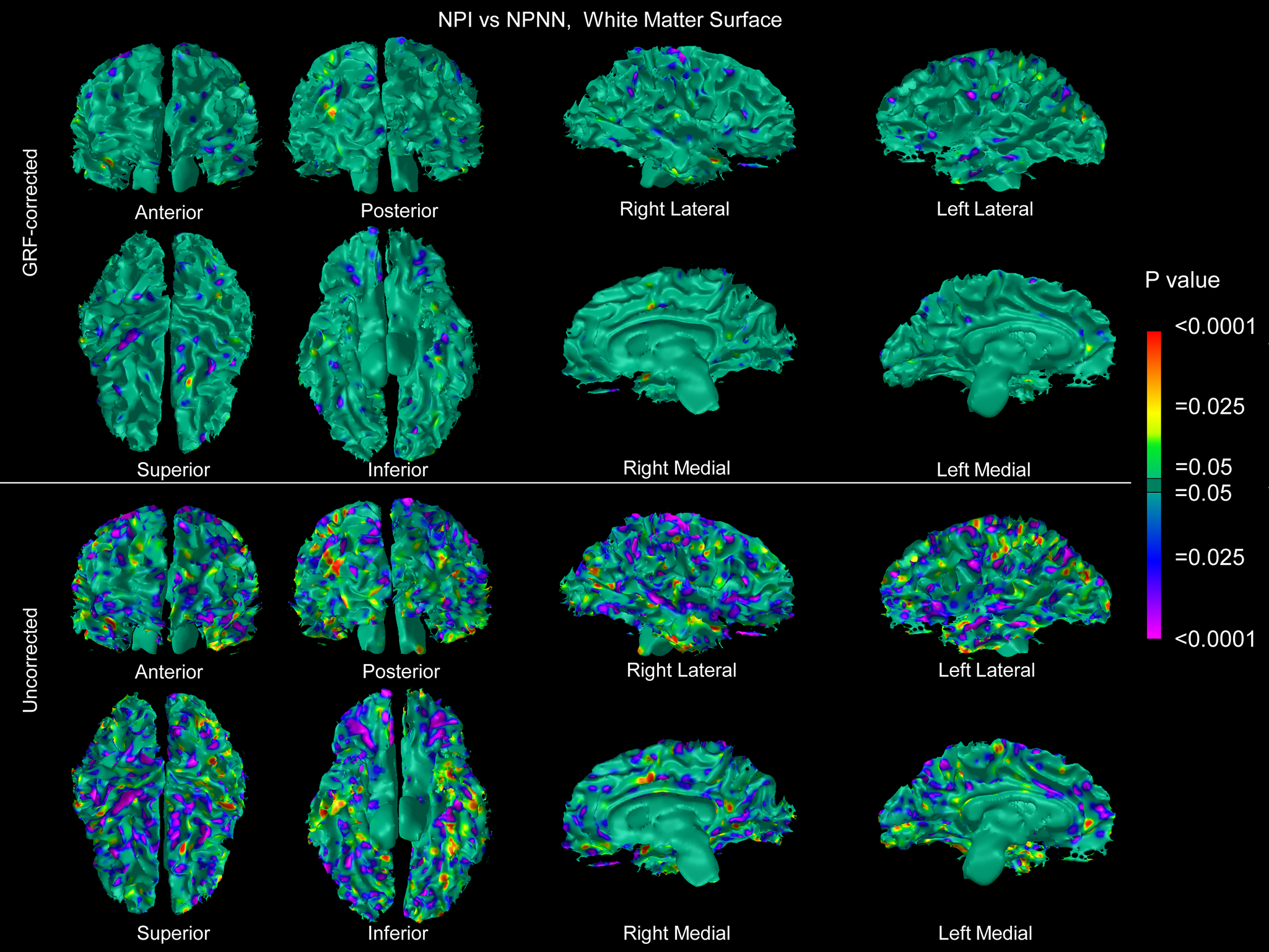

Supplement: Figure S1 — Direct Statistical Comparison of Local Volumes along the Surface of the WM between NPI and NPNN Patients after Covarying for Age, Age2 and Gender. Direct statistical comparisons are depicted between patients with impaired neuropsychological performance (NPI) and patients with near normal neuropsychological performance (NPNN). Statistical effects are color-encoded as in Fig. 1. The figure demonstrates that the NPI subgroup has reduced local volumes of WM along the surface of the WM when compared to NPNN subgroup. The findings survive correction for multiple comparisons. (Upper) Results after GRF- correction. (Lower) Uncorrected results. (TIF) [file pone.0055783.s001.tif]

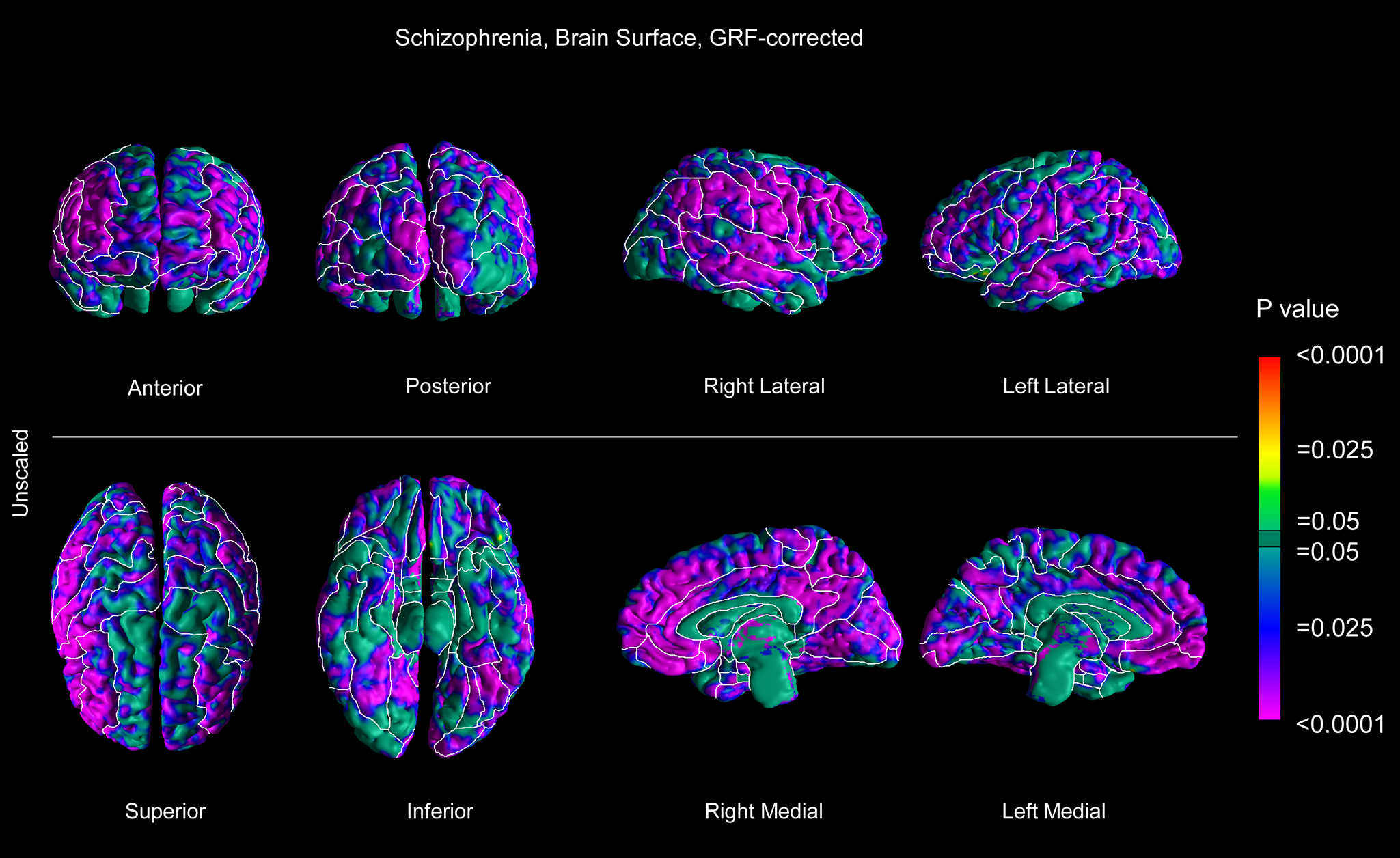

Supplement: Figure S2 — Comparison of Local Volumes along the Surface of the Brain between Persons with Schizophrenia and Healthy Controls without Scaling Correction. Statistical effects are color -encoded as in Fig. 1. Results are GRF-corrected and should be compared with the scaled results for the same comparison in Fig. 1. Briefly, when analyzing unscaled data, we observed, in the patient group, generalized reductions in local volumes of brain tissue along the entire medial and lateral brain surfaces, more prominent in the right than in the left hemisphere. This figure shows that when we do not correct for whole brain volume, patients with schizophrenia exhibit overall smaller cerebral volume than healthy controls. The comparison between scaled and unscaled cortical thickness data did not detect significant differences (not shown), likely because the scaling relationship of brain surface with overall brain size was greater than that of cortical thickness with overall brain size. (TIF) [file pone.0055783.s002.tif]

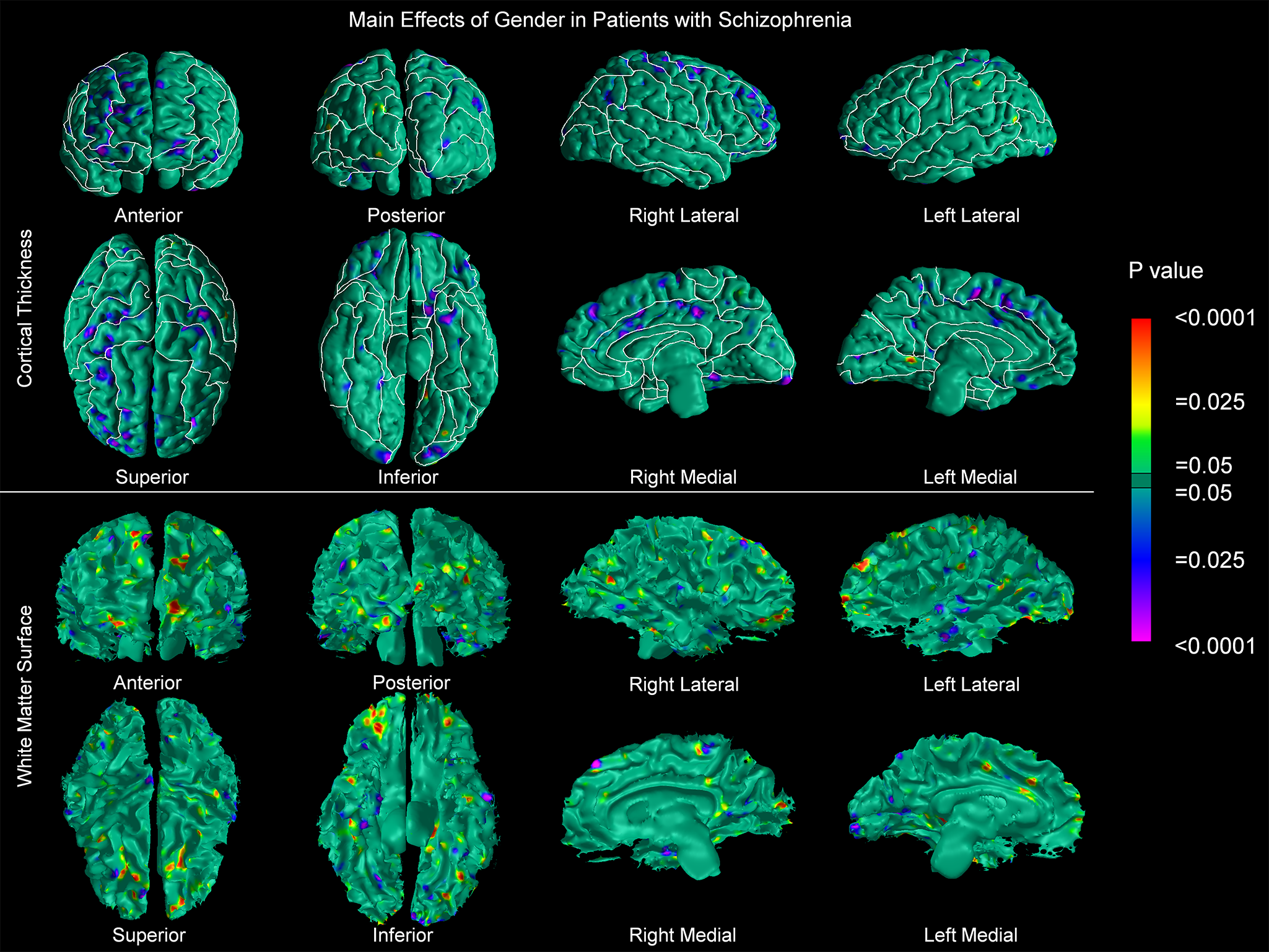

Supplement: Figure S3 — Main Effects of Gender on Cortical Thickness (Top) and on Local Volumes along the Surface of the WM (Bottom) in Patients with Schizophrenia. Statistical effects are color -encoded as in Fig. 1. Results are GRF-corrected. This figure shows greater local volumes along the surface of the WM in male patients relative to female patients, mostly in the superior frontal gyrus. Males also show thinner cortices in the same location compared with females. These results are consistent with our quantitative finding that male patients exhibit larger total surface area of the WM than female patients. A similar pattern has been previously observed both in patients and in healthy control samples. We do not report effects in our healthy control group, which consisted mainly of small changes in local volumes along the surface of the WM in the left hemisphere, because these findings were much less significant and therefore of difficult interpretation. (TIF) [file pone.0055783.s003.tif]

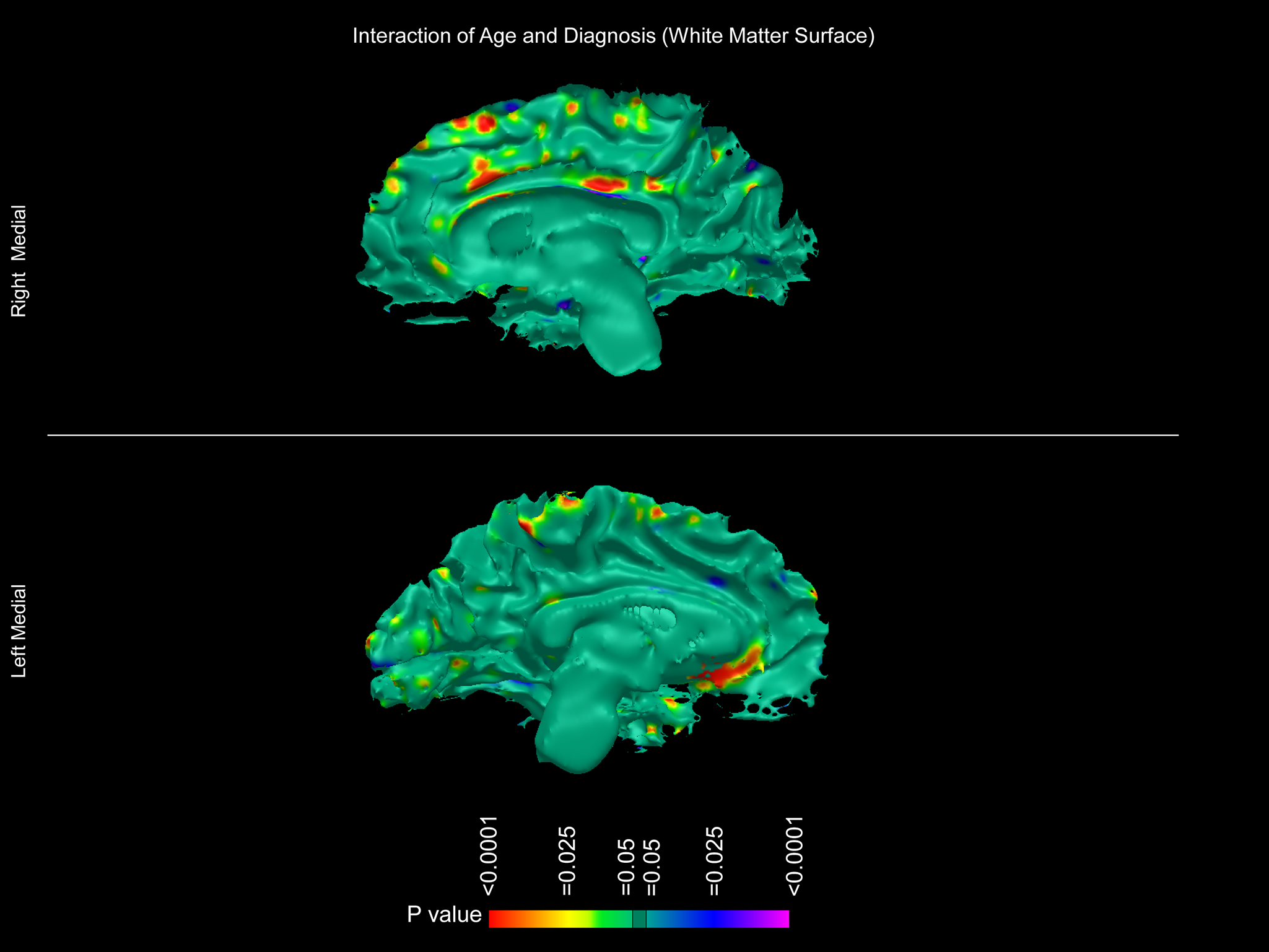

Supplement: Figure S4 — Age by Diagnosis Interactions. Local Volumes along the Surface of the WM of the Right Hemisphere ( Top ) and of the Left Hemisphere ( Bottom ). Statistical effects are color -encoded as in Fig. 1. Results are GRF-corrected. This figure shows greater local volumes along the surface of the WM of the anterior cingulum (underlying the ACC) associated with age, as well as correspondingly greater local volumes along the surface of the brain. No interaction effects on cortical thickness were observed. The interaction term captures the different relationship of WM with age in the healthy controls and in the patient group. This interaction term is significant in voxels different than those where the main effects of diagnosis were observed, and it is highly significant in the cingulum just anterior to the genu of the corpus callosum. Plotting the relationship between age and anatomical measures (not reported) allowed us to conclude that this interaction reflected greater local WM volumes along the surface of the WM of the anterior cingulum in progressively older patients. Conversely, healthy controls exhibited progressively smaller local volumes with increasing age in this same region. This increase notwithstanding, when patients are compared to healthy controls, they do show diffuse reductions in WM (Fig. 1). We do not report the main effects for age and age2 because they were minimal and thus of difficult interpretation. Briefly, they consisted in a very small increase in local volumes at the WM surface of the superior frontal gyrus as well as a decrease in the same area associated with its quadratic term (age2). The combination of these effects followed an inverted U curve. This pattern was identical at the brain surface. No abnormalities in cortical thickness were observed. (TIF) [file pone.0055783.s004.tif]

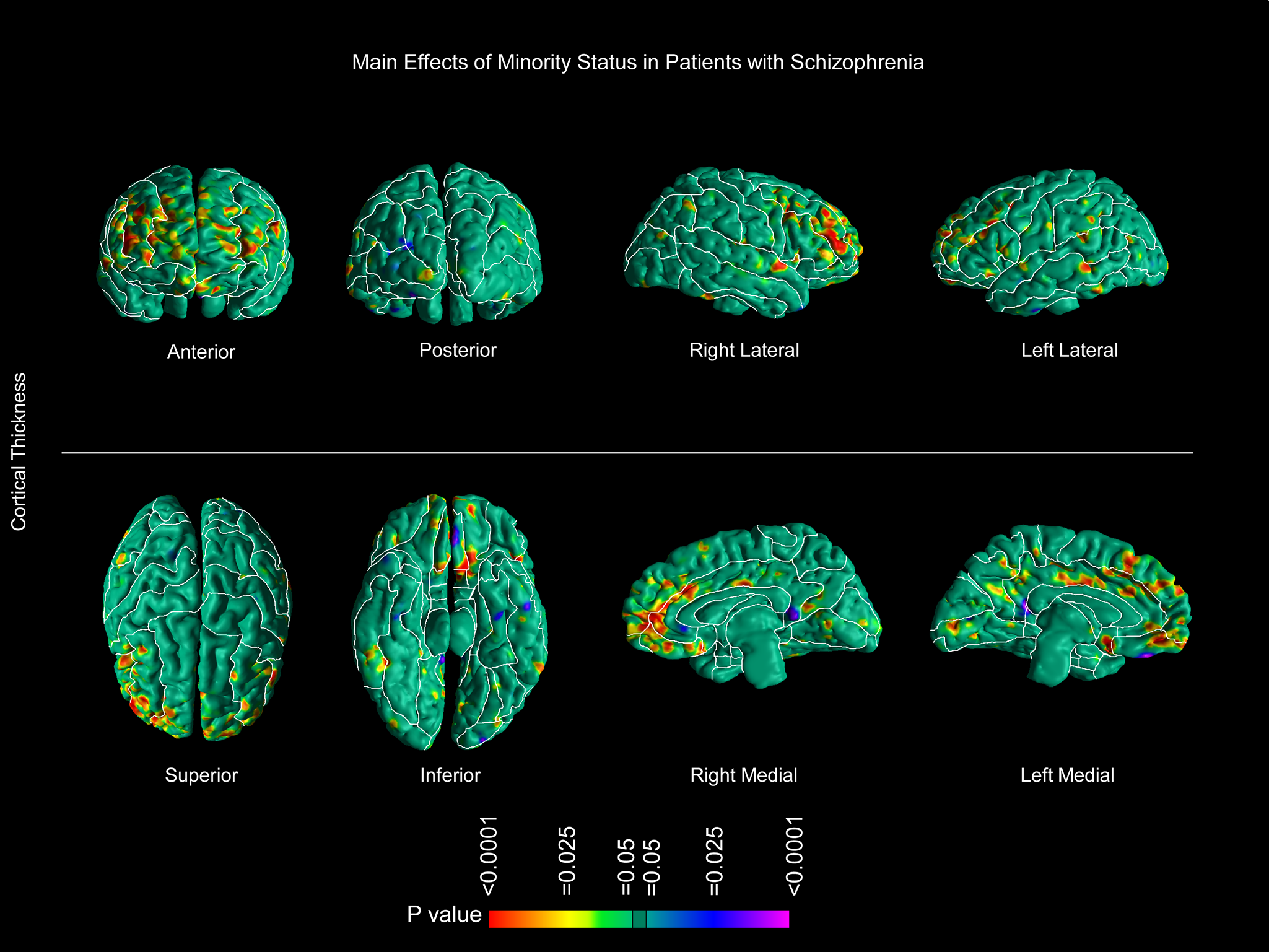

Supplement: Figure S5 — Main Effects of Minority Status on Cortical Thickness in Patients with Schizophrenia. Statistical effects are color encoded as in Fig. 1. Results are GRF-corrected. Region definition was not applied. This figure shows correlations of measures of cortical thickness with minority status. Minority status was defined for the purposes of this analysis as being non-Caucasian. Minority patients exhibit thicker cortices in the superior and middle frontal gyri as well as along the cingulate gyri. Although the effect of minority group position has already been reported in psychotic syndromes, the specific meaning of increased cortical thickness in our sample is unclear, but perhaps related to possible differential environmental exposures. Main effects for minority status were not observed in the healthy control group. (TIF) [file pone.0055783.s005.tif]
