# Supplementary material for: Anatomical Abnormalities in Gray and White Matter of the Cortical Surface in Persons with Schizophrenia
Source: PLoS One. 2013 Feb 13;8(2):e55783. doi: 10.1371/journal.pone.0055783 (PMC3572102; doi:10.1371/journal.pone.0055783)
Supplement: Methods S1 — Supplementary Methods. (DOC) [file pone.0055783.s006.doc]

**Methods (Supplementary Material)**

The marching-cubes algorithm generates a surface representation of all voxels with the specified intensity (iso-value) within a volume sampled on a rectangular grid. The algorithm considers a cube of volume defined by eight neighboring voxels positioned at its corners and determines the polygon that represents the surface through that cube. This polygon is fused to the already generated surface to generate the surface through the entire volume. The algorithm assesses whether the intensity of a voxel matches the iso-value to determine whether the cube intersects the surface and guarantees that each triangle lies in the cubic space enclosed by the eight neighboring voxels at the corners of the cube.

**References (Supplementary Material)**

1. Lorensen W, Cline H. (1987) Marching cubes: a high resolution 3D surface reconstruction algorithm. Computer Graphics. 21:163–169.

2. Hao X, Xu D, Bansal R, Liu J, Peterson BS. (2011) An Improved Representation of Regional Boundaries on Parcellated Morphological Surfaces. Computerized Medical Imaging and Graphics. 35:206–219.
